# Inhibitory Receptor Crosslinking Quantitatively Dampens Calcium Flux Induced by Activating Receptor Triggering in NK Cells

**DOI:** 10.3389/fimmu.2018.03173

**Published:** 2019-01-14

**Authors:** Sridharan Ganesan, Petter Höglund

**Affiliations:** ^1^Department of Medicine Huddinge, Center for Hematology and Regenerative Medicine, Karolinska Institutet, Stockholm, Sweden; ^2^Clinical Immunology and Transfusion Medicine, Karolinska University Hospital, Huddinge, Sweden

**Keywords:** NK cells, MHC class I, calcium flux, Ly49, NKG2A, inhibition, IL-15, quantitative

## Abstract

Natural killer (NK) cell function is regulated by a balance between activating and inhibitory receptors, but the details of this receptor interplay are not extensively understood. We developed a flow cytometry-based assay system in which Ca^2+^ flux downstream of antibody-mediated activating receptor triggering was studied in the presence or absence of inhibitory receptor co-crosslinking. We show that the inhibitory influence on activating receptor-induced Ca^2+^ flux is quantitatively regulated, both on murine and human NK cells. Furthermore, both activating and inhibitory receptors operate in an additive way, suggesting that a fine-tuned balance between activating and inhibitory receptors regulate proximal NK cell signaling. We also demonstrate that murine NK cell expression of H2D^d^ lowered the capacity of Ly49A to deliver inhibitory signals after antibody crosslinking, suggesting that the *cis* interaction between H2D^d^ and Ly49A reduce the signaling capacity of Ly49A in this setting. Finally, we show that priming of NK cells by IL-15 rapidly augments Ca^2+^ flux after activating receptor signaling without attenuating the potential of inhibitory receptors to reduce Ca^2+^ flux. Our data shed new light on NK cell inhibition and raises new questions for further studies.

## Introduction

Natural Killer (NK) cells are innate lymphocytes involved in the immune defense against viral infections and transformed cells (1), and they also exert control over adaptive immune responses (2, 3). NK cells mediate killing of target cells through the secretion of perforin, granzymes and the interaction of Fas-Fas-ligands (4, 5), and they also secrete a multitude of cytokines with immunomodulatory properties (6). The effector functions of NK cells are regulated by signals coming from activating and inhibitory cell surface receptors, which are integrated inside the cells to determine the functional output (7, 8).

In contrast to T cells, each of which possess a unique T cell receptor generated by somatic gene recombination, NK cells express several distinct types of germline-encoded activating receptors that are differentially expressed, signal differently and bind unique ligands on target cells (9). A similar diversity can be found for inhibitory receptors for MHC class I. In mice these belong to the lectin-like Ly49 receptor family, whereas in humans they constitute immunoglobulin superfamily killer immunoglobulin-like receptors (KIR) (10). Both mice and humans also express NKG2A, a lectin-like inhibitory receptor for non-classical MHC class I molecules (11, 12). Ly49 and KIR receptors, respectively, are expressed on overlapping NK cell subsets, in an as yet unexplained manner creating an “NK cell repertoire,” characterized by NK cells expression from zero up to at least 5 inhibitory receptors (13).

During interactions with MHC class I molecules on target cells, inhibitory receptors exert a negative influence on NK cell activation by the recruitment of phosphatases, in particular SHP-1 and SHIP, resulting in diminished NK cell activating receptor signaling (14). The mechanisms underlying NK cell inhibition is complex, but the most studied mechanism involves a de-phosphorylation event acting on the intracellular signaling mediator VAV, which act downstream of activating NK cell receptors (15, 16). Furthermore, a recent study showed that inhibitory receptor engagement increased ubiquitilation of LAT, suggesting that inhibitory receptor signaling may also function by limiting the availability of signaling substrates inside the NK cells (17).

Given the central role for inhibitory receptors in NK cell function, surprisingly little is known about the nature of the activating and inhibitory signal cross-talk in NK cells. It is assumed that intracellular signals transduced by the triggering of activating and inhibitory receptors are integrated through a cross-talk proximal to the cell membrane (8, 17, 18), but this cross-talk has not been studied in much detail. In addition, while it is suggested that NK cell activation is controlled by a threshold of activation (19), it is not clear whether a signaling threshold can be measured experimentally at the level of early Ca^2+^ flux. Furthermore, whether or not the inhibitory signals are binary (all or none) or dynamic and quantitative, if they are affected by the *cis* interaction with MHC class I or if they are influenced by cytokines that regulate NK cell function, such as IL-15, are remaining questions.

We developed an assay to measure the inhibitory influence by Ly49 or NKG2A receptors on murine NK cell activation and by the NKG2A receptor on human NK cells, read out as the inhibition of Ca^2+^ flux after co-crosslinking of activating receptors. Intracellular Ca^2+^ fluxes correlate with NK cell effector functions, including degranulation and cytokine production (20). Using this assay, we provide several novel insights of relevance to the way by which inhibitory receptors may control NK cell function.

## Results

### Activating Receptor Crosslinking Quantitatively and Additively Modulates Ca^2+^ Flux in Primary Mouse NK Cells

It has been demonstrated that inhibitory receptor ligation exert proximal down-modulatory effects on signaling pathways downstream of activating receptors, but the nature of these inhibitory influences have not been extensively studied. To gain further insight into this process, we established an *in vitro* system based on co-crosslinking of activating and inhibitory NK cell receptors on mouse and human NK cells, followed by a FACS-based assay for Ca^2+^ flux in real time (Figure S1). We reasoned that this setup would allow us to investigate if inhibitory receptor triggering quantitatively downregulates NK cell activation, or if inhibition would operate in a threshold mode.

**Figure 1:**
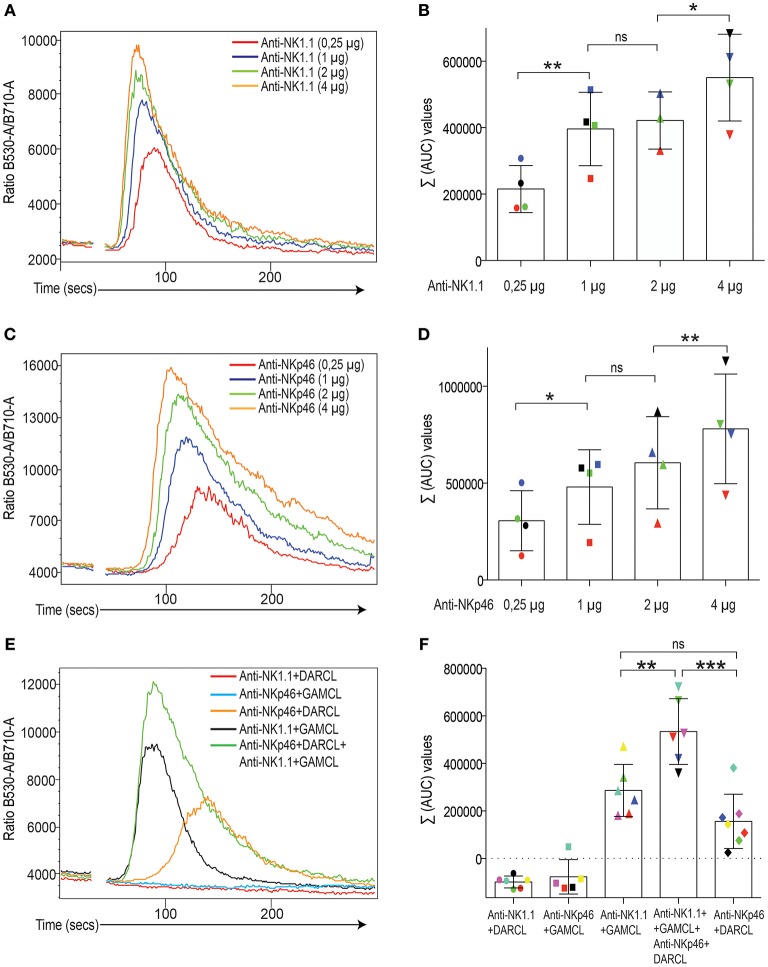
. Induction of Ca^2+^ flux in mouse NK cells after crosslinking of activating receptors. **(A)** Calcium flux response (kinetics plot of the ratio between Fluo-4 and Fura-red) after NK1.1 crosslinking. The colored lines depict various concentrations of the primary antibody.One representative experiment. **(B)** Total area-under-the-curve (AUC) values after baseline correction (see Materials and Methods) from four independent experiments. Different colors indicate different experiments. Statistics calculated using a one-way paired Student's *T*-test. **(C)** Same setup as in A but with an antibody against NKp46. **(D)** Same setup as in **(B)** but for NKp46 antibody. **(E)** Calciums flux response (kinetics plot of the between Flou-4 and Fura-red) after individual and simultaneous crosslinking of NK1.1 and NKp46 on isolated NK cells. Different crosslinkers were used for NK11 and NKp46 primary antibodies. DARLC, donkey-anti-rat (used for NKp46) and GAMCL, goat-anti-mouse (used for NK1.1). One representative experiment. Note lack of cross-reactivity beteen the crosslinkers (flat lines). **(F)** Total AUC values after baseline correction (see Material and Methods) from 5 to 7 independent experiments, which are color-coded. Some experiments did not inluce all groups. Note additive Ca2+ flux response when two receptors were crosslinked simultaneously. Statistical analysis was performed using a one-way ANOVA with Tukey's multiple comparisons test. ^*^p < 0.05, ^**^p < 0.01, ^***^p < 0.001, ns, not significant.

In a first step, we identified reagents that could be used to identify subsets of mouse NK cells and at the same time be used to cross-link activating and inhibitory receptors simultaneously (Table S1). Following crosslinking of NK1.1-APC-stained NK cells using a F(ab)_2_ fragment of a goat-anti-mouse secondary antibody, a flux of Ca^2+^ characterized by a rapid onset, a peak and a relaxation phase was recorded in real-time using a ratiometric flow cytometry method based on Fluo-4 and Fura-Red staining (Figures 1A,B; see Materials and Methods). Crosslinking the activating receptor NKp46 also elicited a rapid Ca^2+^ flux response in mouse NK cells (Figures 1C,D), but with different kinetics characterized by a slower onset compared to NK1.1. For both NK1.1 and NKp46 stimulation, the extent of Ca^2+^ flux was sensitive to the amount of primary antibody in all experiments, at least for the concentrations of crosslinking antibodies we used (Figures 1B,D). In line with a quantitative response to signaling strength, when these two activating receptors were co-crosslinked, NK cells displayed an additive enhanced calcium flux response, with both earlier onset and higher peak value in NK cell crosslinked via the two receptors simultaneously (Figures 1E,F).

### Inhibitory Receptor Crosslinking Quantitively and Additively Downregulates Activating Signals in Primary Mouse NK Cells

We next tested if co-crosslinking NK1.1 and NKp46 receptors with inhibitory Ly49 or NKG2A receptors simultaneously would dampen Ca^2+^ flux triggered by the activating receptors, which would support a proximal influence on early NK cell signaling. To test this, we designed a co-staining protocol in which NK cells were double-stained with antibodies against activating receptors NK1.1 or NKp46 and antibodies against either Ly49A, Ly49G2, or NKG2A, with the aim of co-crosslinking these antibodies using an IgG F(ab)_2_ fragment with specificity for both activating and inhibitory antibodies. In order for this setup to work as intended, it was necessary to make sure that flourochrome-labeled primary antibodies against inhibitory receptors could be used (Figure S1). Because all inhibitory receptors in the mouse are expressed on subsets of NK cells, this setup would allow detection of a control subset and a test subset in the same sample, providing a critical test if inhibitory receptor triggering quantitatively downregulates NK cell triggering, or if it operates by means of a threshold effect.

**Figure 2:**
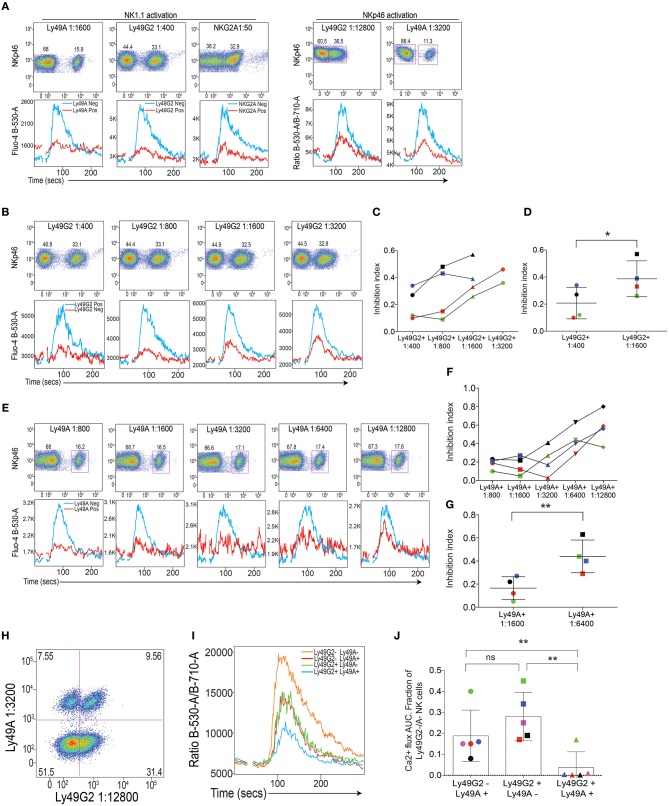
Inhibitory receptor (IR) crosslinking quantitatively downmodulates Ca^2+^ flux induced by activating receptor stimulation. **(A)** Top: Staining of Ly49A, Ly49G2 and NKG2A on mouse NK cells. Bottom: Kinetics plot of Fluo-4 fluorescence after crosslinking of NK cells coated with antibodies against NK1.1 (left) and NKp46 (right) in the presence of antibodies against inhibitory receptors Ly49A, Ly49G2 and NKG2A as indicated. Red line represents the response on the NK cell subset expressing the inhibitory receptor in question and the blue line represents the inhibitory receptor-negative subset in the same sample. Gates were set on plots in the top panel. Corresponding concentrations of the antibodies in the various dilutions are indicated in Table S1. **(B)** Reduction of inhibitory influence on activating signals after titration of inibitory antibody. Top: staining of Ly49G2 at various antibody dilutions. Bottom: Kinetics plot of Fluo-4 fluorescence after NK1.1 activation in the presence of various concentrations of Ly49G2. Red line represents the response on the NK cell subset expressing the inhibitory receptor in question and the blue line represents the inhibitory receptor-negative subset in the same sample. **(C)** The “inhibition index” (see Materials and Methods) becomes bigger following reduced concentration of inhibitory recept antibody. Data from four individual color-coded experiments. The red experiments is the one depicted in **(B)**. **(D)** Statistical analysis between the 1:400 and 1:1600 groups in **(C)** using Student's *T*-test. **(E–G)** Same setups as in **(B–D)** but for Ly49A instead. **(H)** Double staining of Ly49A and Ly49G2 on isolated mouse NK cells. Dilutions of the antibodies was chosen so as to provide clearly detectable stainings an at the same time only partial inhibition. **(I)** Kinetics plot of the Fluo-4/Fura-red flourescence ratio in NK cells expressing Ly49A, Ly49G2, both receptors or none of these receptors. **(J)** Statistical analysis of the AUC values after baseline correction of Ca^2+^ flux from the three subsets expressing either one or both inhibitory receptors in I. Summary of 5 independent experiments, which are color-coded. The red experiment is shown in **(I)**. Statistical analysis in **(D,G)** was performed using a two-way Student's T test and in **(J)** using a one-way ANOVA with Tukey's multiple comparisons test. ^*^p < 0.05, ^**^p < 0.01, ns, not significant.

When applying this setup for Ly49A, Ly49G2, and NKG2A, we confirmed our previous data (21), showing that these receptors were all capable of downregulating Ca^2+^ flux triggered by NK1.1 simulation (Figure 2A, left). Two of the inhibitory receptors, Ly49A and Ly49G2 were also tested in conjunction with NKp46, with similar outcome (Figure 2A, right). By titrating the amount of antibody against the inhibitory receptors, we established that both Ly49A and Ly49G2 crosslinking downregulated ITAM-dependent NK1.1 and NKp46 triggering in a dose-dependent way, suggesting that the inhibitory receptor activity, at least in this setup, was able to quantitatively inhibit Ca^2+^ flux downstream of activating receptor ligation (Figures 2B–G). NKG2A was not possible to use in these titration experiments, since staining was rapidly lost after titration and the cells could not be identified clearly using lower concentrations than 1:50.

NK cells express combinations of inhibitory receptors, some of which may be specific for the same MHC class I allele (13). Thus, it was of interest to ask if two different inhibitory receptors would co-operate for inhibition of ITAM signaling, which was previously suggested in assays of NK cell cytotoxicity (7). To test this, we stained NK cells with antibodies against NKp46, Ly49A, and Ly49G2 (all three being rat antibodies), and added donkey-anti-rat F(Ab)_2_ fragments for crosslinking. By gating on NK cells based on either one or both inhibitory receptors (Figure 2H), we asked if crosslinking of two receptors would inhibit Ca^2+^ flux more efficiently than co-crosslinking of only one inhibitory receptor? Our experiment with this setup demonstrated that NK cell expressing both inhibitory receptors indeed showed less Ca^2+^ flux compared to NK cells expressing only one inhibitory receptor (Figures 2I,J), suggesting that two inhibitory receptors co-operate in limiting proximal NK cell signaling downstream of ITAM signaling.

### The Inhibitory Effect of Ly49A, but Not Ly49G2, Is Reduced by Expression of H2D^d^ at the Cell Surface of the Ly49A^+^ NK Cell

**Figure 3:**
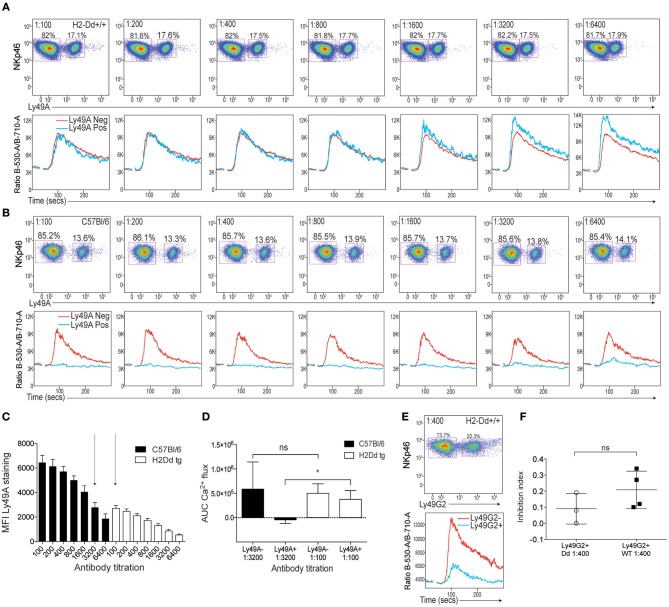
Poor effect of Ly49A receptor crosslinking on NK1.1-induced Ca^2+^ flux in NK cells from mice expressing the Ly49A ligand H2D^d^. **(A)** Top: Staining of Ly49A on NKp46^+^ NK cells from H2D^d^ mice at various dilutions of anti-Ly49A antibodies. Bottom: Kinetics plot of the Fluo-4/Fura-red flourescence ratios in NK cells from H2D^d^ mice stained with various concentrations of anti-Ly49A together with a fixed concentration of anti-NK1.1. The blue line represents the response of the NK cell subset expressing Ly49A and the red line represents Ly49A-negative NK cells in the same sample. **(B)** Same setup as in A but on NK cells from B6 mice. **(C)** Mean fluorescence intensity (MFI) of Ly49A staining at various dilutions of anti-Ly49A on NK cells derived from B6 (black bars) or H2D^d^ (white bars) mice. Three independent experiments are pooled. Arrows indicate antibody titrations at which the MFI of Ly49A staining was similar between B6 and H2D^d^ mice. **(D)** AUC values of Ca^2+^ flux in Ly49A-positive and Ly49A-negative NK cells from B6 and H2D^d^ mice at the anti-Ly49A concentrations indicated by arrows in **(C)** (1:100 for H2D^d^ and 1:3,200 for B6). Statistical analysis performed using one-way ANOVA with Tukey's multiple comparisons test. **(E)** Top: Staining of Ly49G2 on NKp46^+^ cells from H2D^d^ mice. Bottom: Kinetics plot of the Fluo-4/Fura-red flourescence ratios in Ly49G2-positive (blue) and Ly49G2-negative (red) NK cells from H2D^d^ mice stained with anti-Ly49G2 at 1:400 dilution. **(F)** Inhibition index comparison between the degree of Ly49G2-mediated inhibition in H2D^d^ mice and B6 mice. Based on 3–4 independent and comparable experiments. Statistics was calculated using a paired Student's *T*-test. ^*^p < 0.05, ns , not significant.

Several studies have reported that the Ly49A receptor binds its ligand H2D^d^ both in *cis*, i.e., on the same cell, and in *trans*, i.e., when the ligand is expressed by surrounding cells. This binding is mediated by the same interaction site and the two modes of binding appear to be mutually exclusive (22, 23). Thus, *cis* interaction with H2D^d^ restricts access of the Ly49A receptor to H2D^d^ presented in *trans*, e.g., in the form of H2D^d^ tetramers (24). To provide further insight into the inhibitory function of the Ly49A receptor on NK cells expressing H2D^d^, we performed crosslinking experiments of Ly49A on NK cells from mice expressing H2D^d^ as a single MHC class I molecule and compared the results with crosslinking of Ly49A on NK cells from B6 mice, which lack H2D^d^. Despite the fact that Ly49A^+^ NK cells are more functional in H2D^d^ mice compared to B6 mice (25), no inhibitory function of Ly49A was seen at any antibody concentrations that were tested (Figure 3A). In contrast, even the lowest dilution of anti-Ly49A antibody tested (1:6,400) yielded strong inhibition of B6 NK cells in this series of experiments (Figure 3B).

The *cis* interaction with H2D^d^ restricts cell surface expression of Ly49A by a combination of Ly49A internalization and antibody epitope blocking (26). It was therefore possible that the lack of inhibition of Ly49A^+^ NK cells from H2D^d^ mice was the result of lower amounts of Ly49A antibody at the surface of H2D^d+^ NK cells compared to B6 NK cells. To control for this, we performed a parallel titration of antibody staining on H2D^d^ mice and B6 mice and related the inhibitory capacity of the antibody to the cell surface staining intensity. When we compared samples from B6 and H2D^d^ mice that showed the same intensity of anti-Ly49A staining, 1:3,200 in B6 vs. 1:100 in H2D^d^ mice (Figure 3C), we found a clear inhibitory effect on B6 NK cells but no inhibition at all on H2D^d^ NK cells (Figure 3D), supporting a qualitatively different role of the Ly49A receptor to transmit inhibitory signals in the presence of the ligand expressed in the same cell membrane.

To test if the lack of inhibitory function was unique to the Ly49A receptor, we crosslinked the Ly49G2 receptor on H2D^d^ and B6 NK cells and observed clear inhibition of Ca^2+^ flux on both H2D^d^ and B6 NK cells (Figures 3E,F). Thus, on H2D^d^ NK cells, the poor accessibility and poor signaling capacity seems unique to the Ly49A receptor and does not apply to the Ly49G2 receptor, which is consistent with poor ability of Ly49A to bind H2D^d^ in trans (26). These data suggest that the *cis* interaction not only restricts binding, but also the ability of the receptor to deliver inhibitory signals into the cell after antibody-mediated crosslinking at the cell surface.

### NK Cell Priming by IL-15 Does Not Influence the Inhibitory Capacity of Ly49 Receptors

**Figure 4:**
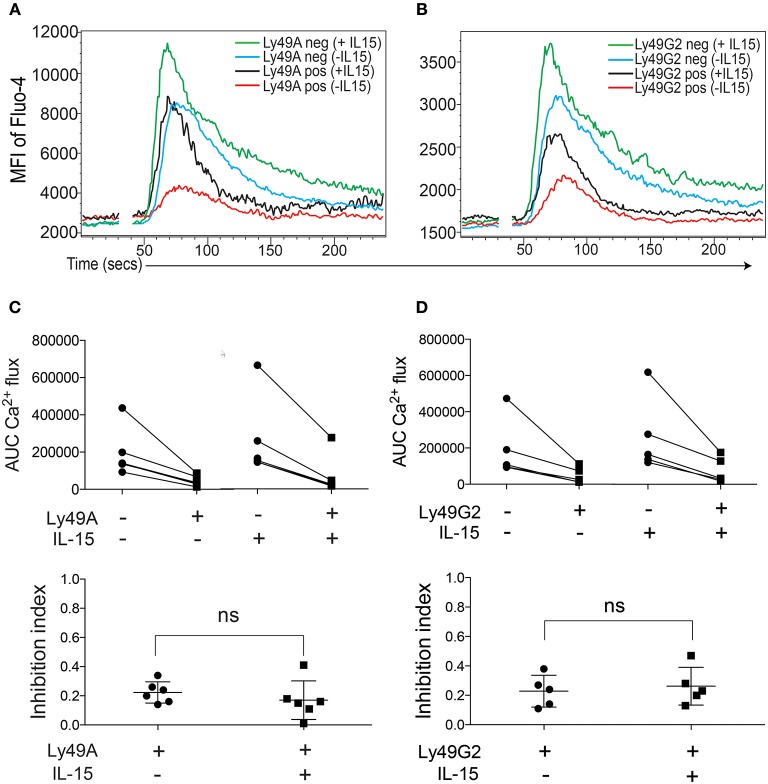
IL-15 priming does not abrogate the capacity of inhibitory receptor crosslinking to dampen Ca^2+^ flux in B6 NK cells. **(A)** One representative experiment showing kinetics of Fluo-4 fluorescence in Ly49A^+^ and Ly49A^−^ IL-15-primed or non-primed NK cells. **(B)** Same as in **(A)** but gated on Ly49G2^+^ NK cells. **(C)** Top: Total AUC values after baseline correction of Ly49A^−^ and Ly49A^+^ NK cells with or without IL-15 from 6 independent experiments. Lines connect subsets from the same experiment. Bottom: Inhibition index for anti-Ly49A crosslinking in IL-15-primed and non-primed NK cells. **(D)** Same setup as as in **(C)** but for Ly49G2^+^ NK cells. ns , not significant.

Stimulation with the cytokine IL-15 “primes” NK cells, resulting in a more efficient and more rapid response to activation (27, 28). The mechanisms underlying NK cell priming are not known in detail, but seem to involve an augmentation of the AKT-mTOR axis of NK cell signaling (29–31). Because the purpose of NK cell priming would be to enhance NK cell responsiveness, it was of interest to ask if the capacity for NK cell inhibition would be reduced, or even abrogated, by IL-15-priming. To determine if our experimental system could be used to test this question, we first investigated the Ca^2+^ flux response to NK1.1 triggering in IL-15-primed and non-primed NK cells. A 2-h incubation with IL-15 enhanced NK1.1-triggered Ca^2+^ responses (Figures 4A,B, compare blue and green lines) indicating that IL-15 potentiates ITAM signaling downstream of NK1.1.

When NK1.1 was co-crosslinked with either Ly49A or Ly49G2, we found that IL-15 priming induced Ca^2+^ flux also in the Ly49A^+^ and Ly49G2^+^ NK cell subsets (Figures 4A,B, compare red and black lines), and that the efficiency of the inhibitory receptor to shut off NK1.1-triggered Ca^2+^ release seemed to be unaffected (Figures 4A,B). Quantification of this response in a larger series of experiments substantiated this conclusion, and showed that the efficiency of inhibitory signaling downstream of Ly49A and Ly49G2 was equally efficient on IL-15-primed and non-primed NK cells (Figures 4C,D).

### NKG2A Co-crosslinking Quantitatively, but Variably, Dampens NKp46-Triggered Ca^2+^ Flux in NK Cells From 6 Human Donors

**Figure 5:**
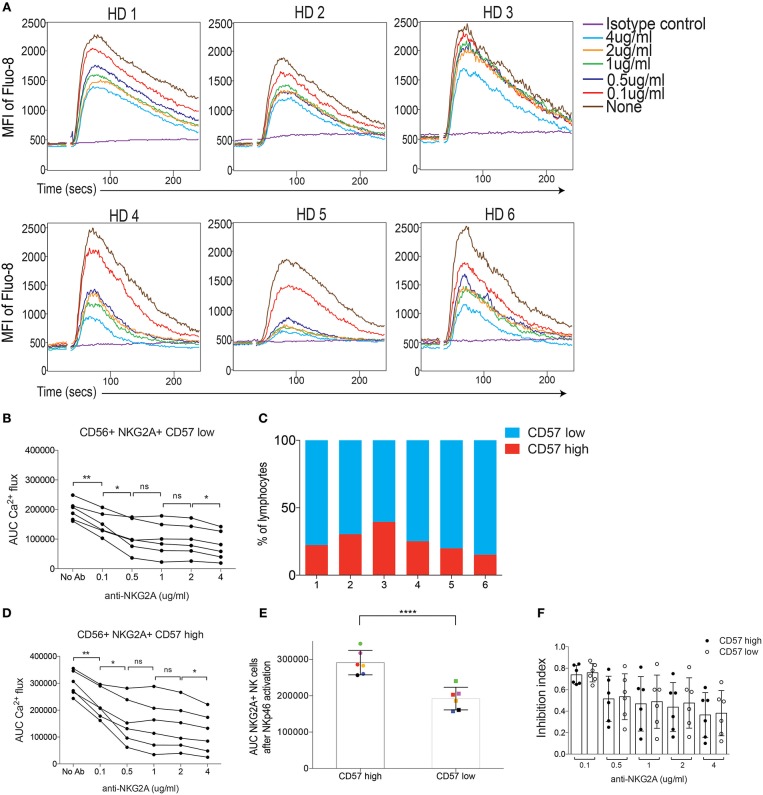
Crosslinking of NKG2A dampens Ca^2+^ flux triggered by NKp46 stimulation in human 6 human donors. **(A)** Kinetics plot of Fluo-8 fluorescence of CD57 low, CD56^+^, NKG2A^+^ NK cells stained with biotinylated anti-NKp46 and biotinylated anti-CD94 and co-crosslinked using by Streptavidin. HD1-HD6 depict the 6 donors. Different concentrations of anti-NKG2A are color-coded. **(B,D)** Total AUC values of CD56^+^ NKG2A^+^ CD57 low NK cells, after baseline correction, of the data in **(A)**. Statistics were calculated using a paired Student's *T*-test for the combinations indicated. **(C)** The fractions of CD57high/CD57low NK cells from HD1-HD6. Gating as indicaed in Figure S2. **(D)** Similar data as in **(B)** but for CD56^+^ NKG2A^+^ CD57 high NK cells. **(E)** Total AUC values of CD57 high and CD57 low NK cells stimulated with biotinylated anti-NKp46 and crosslinked with Streptavidin, in the absence of inhibitory receptor crosslinking. Statistics were calculated using a parid Student's *T*-test. **(F)** The inhibition index obtained by streptavidin-mediated crosslinking of NKG2A^+^ NK cells at lowering concentration of biotinylated anti-CD94, and comparing CD57 low and CD57 high NK cells. No significant differences were seen. ^*^p < 0.05, ^**^p < 0.01, ^****^p < 0.0001, ns, not significant.

We next investigated if inhibitory signaling also dampened Ca^2+^ flux downstream of activating receptors in human NK cells. Here, we focused on the NKG2A receptor and used streptavidin to co-crosslink biotinylated antibodies against NKp46 and CD94, using fluorescent antibodies against NKG2A and NKG2C to distinguish these subsets (Figures S2, S3; Table S2). Quantitative inhibition of NKp46-induced Ca^2+^ flux was observed in all 6 donors, albeit with varying efficiency between donors (Figure 5A). Because the CD57 marker was included in the staining protocol (Figure S2) we were able to extend the analysis in human NK cells by gating on this marker, which is frequently used to identify mature NK cells in humans (32). In a first step, we quantified the extent of inhibition on CD57 low NK cells in each donor by calculating the AUC for for each concentration of anti-CD94 antibody. This analysis demonstrated a similar titration curve for each donor, characterized by an early linear drop of varying degree followed by a plateau as the concentreation of anti-CD94 reached 0.5 μg/ml (Figure 5B). Fractions of CD57^high^ NK cells varied between the donors (Figure 5C). When we repeated the same analysis on CD57^high^ NK cells, we found a similar pattern of titration (Figure 5D) but with generally higher AUC values (Figure 5D). When the Ca^2+^ flux after NKp46 crosslinking (in the absence of inhibition) was compared, we found that CD57^high^ NK cells showed a statistically verified stronger Ca^2+^ flux response compared to CD57^low^ NK cells (Figure 5E), suggesting an intrinsically increased responsiveness of the CD57^high^ subset. However, despite this, the inhibitory effect of anti-CD94 crosslinking on NKG2A^+^ NK cells titrated in a similar way in CD57^low^ and CD57^high^ NK cells (Figure 5F), arguing that despite higher responsiveness to activating receptor crosslinking in CD57^high^ NK cells (Figure 5E), the inhibitory capacity of NKG2A was the same (Figure 5F).

## Discussion

We here report that co-crosslinking activating and inhibitory receptors using antibodies resulted in downregulation of Ca^2+^ flux in both mouse and human NK cells in a manner that was sensitive to the amount of added crosslinker against the inhibitory receptor. Similarly, in humans, inhibitory capacity of NKG2A also titrated with the antibody concentration, was variable between donors but independent from the expression of CD57. We also show that activating and inhibitory receptors of different types operated additively, suggesting that they can co-operate in regulating NK cell activity when NK cells meet target with several types of ligands for activating and inhibitory receptors. In the mouse, the inhibitory signaling capacity of the Ly49A receptor was impaired by the presence of H2D^d^ in the same cell, suggesting that the *cis* interaction between H2D^d^ and Ly49A impairs the ability of Ly49A to transmit inhibitory signals after antibody crosslinking. Furthermore, 2 h of IL-15 priming of NK cells augmented Ca^2+^ signaling downstream of the activating receptor NK1.1. Nonetheless, the inhibitory receptor signaling capacity was not changed following IL-15 stimulation. Thus, our assay for inhibitory receptor activity, in which Ca^2+^ release could be studied in inhibitory receptor-positive NK cells in the same sample, has revealed features of inhibitory receptor function that was not previously well characterized.

It is well established that inhibitory receptors are sensitive to different amount of target cell MHC class I, both during NK cell killing (19) and during NK cell education (33–36). To our knowledge, our data is the first, however, to demonstrate that inhibitory receptor signaling quantitatively downmodulates activating receptor signaling toward Ca^2+^ flux, most compellingly demonstrated by the successive decrease in Ca^2+^ flux after NK1.1 or NKp46 receptor co-crosslinking in the presence of increasing amount of antibody, or streptavidin, against inhibitory receptors in two different species. An alternative to this result would have been a threshold of inhibition, which was implied from previous work on NK cell cytotoxicity against target cells transfected with different amount of HLA class I ligands (19). In such a model, inhibition of signaling would require a certain amount of SHP-1 to be recruited to the immune synapse to accumulate close to the activating receptor signaling complex, before a cascade of de-phosphorylation is initiated. Our data argue against such a model and instead suggests a fine-tuned regulation, in which small perturbations in the balance between kinases and phosphatases modulate the threshold of activation for individual NK cells, both in humans and in mice, in real time (37). However, it is likely that the regulation of effector functions such as cytotoxicity or cytokine release are subject to more complex regulation downstream of Ca^2+^ flux, e.g., regulating gene transcription, cellular polarization and granule release necessary for their execution. Activation thresholds may be set at such levels, in a process by which a linear signaling stream triggered by receptor ligation might be translated into a threshold switch more downstream. Testing such a model would require novel types of analysis systems in which effects of inhibitory receptor ligation downstream of Ca^2+^ flux could be evaluated in real time, something which attains significant technical challenges due to the time delays between initial triggering and e.g., granule release.

Some Ly49 receptors, of which Ly49A is the most studied, interact with its MHC class I ligand in *cis, i.e.*, in the same cell membrane (23, 26, 38, 39). This property impacts in many ways on Ly49A receptor biology, the most significant of which is a conformational change in the Ly49A receptor structure into a bent conformation that protects H2D^d^ binding sites for both antibodies and for H2D^d^ tetramers (22–24). In support of this notion, we found limited access of Ly49A on H2D^d^-expressing NK cells to conformation-specific antibodies in this paper. In addition, we were completely unable to observe any negative impact after co-crosslinking Ly49A with NK1.1 on NK cell expressing H2D^d^, which was in contrast to crosslinking Ly49A on H2D^d^-negative NK cells, in which binding of anti-Ly49A antibodies (titrated to the same extent as in the H2D^d^-positive NK cells) provided inhibitory input. This surprising finding raises questions regarding one of the basic tenets of “missing self” recognition, which is that self-tolerance is determined, at the level of the individual NK cell, by inhibitory interactions between Ly49 receptors and self MHC class I on surrounding cells (40). This would be considered particularly important in NK cells expressing Ly49A in H2D^d^ mice, in which Ly49A-expressing NK cell subsets are the most efficiently educated subsets with the best functional capacity (25). It is important to note, however, that our data does not imply that the Ly49A receptor complexed in *cis* with H2D^d^ is completely unable to provide inhibitory input from H2D^d^ expressed on other cells. Binding of Ly49A to its “real” ligand H2D^d^ on target cells during effector target interactions, might lead to competition between external and internal H2D^d^ that could liberate Ly49A epitopes for H2D^d^ binding on other cells. Arguing against this, on the other hand, is the finding that the switch between *cis* and *trans* conformation of Ly49A is predicted to be energetically unfavorable (22). In the end, this question must be tested experimentally by isolating NK cells from H2D^d^ mice expressing only Ly49A and subject them to lysis of target cells expressing H2D^d^. So far, our system, which measures inhibitory input from single Ly49 receptors, nevertheless implies that the *cis* interaction severely limits the capacity of Ly49A to transmit negative signals to NK cells.

One additional point should be made in conjunction with this discussion. We did, reproducibly, find that lowering the anti-Ly49A concentration paradoxically increased Ca^2+^ flux in Ly49A^+^ NK cells in H2D^d^ mice while at the same time leaving Ca^2+^ flux in Ly49A- NK cell unaffected. We think this finding reflects the higher responsiveness of Ly49A^+^ vs. Ly49A- NK cells in H2D^d^ mice (25, 41), which becomes apparent as the anti-Ly49A antibody is diluted and loses its inhibitory potential, yet remains visible for detection of Ly49A^+^ NK cells using FACS. If this interpretation is correct, it suggests that anti-Ly49A antibodies are capable of triggering some degree of inhibition of Ly49A^+^ NK cells from H2D^d^ mice, when applied at very high concentrations.

The cytokine IL-15 plays a pleiotropic role in NK cell biology, including being a priming factor for NK cell functional capacity (27–29). The priming effect of IL-15 is manifested as an augmentation of functional responses, such as killing and cytokine secretion, as a result from a short incubation time before NK cell function is triggered. Given this apparent direct impact on ITAM-dependent NK cell signaling, it was of interest to evaluate if part of the priming response could involve effects on the inhibitory signaling pathways, perhaps manifested as a reduced inhibitory effect of a given inhibitory receptor trigger. Our data did not support such a role but suggest that IL-15 priming is in direct balance with Ly49 receptor triggering and that no specific impact on the inhibitory receptor signaling components was revealed. A conclusion of the same type could be made when comparing CD57^high^ and CD57^low^ NK cells in human NK cells, which suggest that NK cells displaying an intrinsically higher responsiveness were similarly sensitive to inhibitory signals. Taken together, these data thus propose that the responsiveness of NK cells, dictated by the intrinsic capacity of activating signaling pathways determined e.g., by maturation or priming, do not influence the degree of inhibition by a given inhibitory stimulus.

In summary, our study provide evidence to suggest that inhibitory Ly49 and NKG2A receptors downregulate Ca^2+^ flux downstream of ITAM signaling in a linear way, following co-crosslinking of activating and inhibitory receptors on both human and murine NK cells. Furthermore, our data imply that the *cis* interaction between Ly49A and H2D^d^ strongly reduces the capacity of Ly49A to transmit negative signals after crosslinking and that short-term IL-15 priming operates independently from inhibitory receptor signaling. New experiments will be required to elucidate the molecular basis for these findings.

## Materials and Methods

### Mice

C57Bl/6 (B6) mice were purchased from Jackson laboratory and were used at 6–10 weeks of age. Mice transgenic for H2D^d^ (and lacking H2^b^ molecules) were previously described (25, 41) and were bred and maintained in the animal facility at Karolinska Institutet, Huddinge. Breedings and animal handling followed institutional guidelines at Karolinska Institutet and at the Karolinska University Hospital. All experimental procedures were approved by the Stockholm branch of the regional animal ethics committee in Sweden (Stockholm Södra Djurförsöksetiska Nämnd and Linköpings Djurförsöksetiska Nämnd).

### Antibodies and Flow Cytometry Analysis

All antibodies and their concentrations used in this study are detailed in Tables S1, S2. For flow cytometry, single cell suspension of mouse splenocytes were incubated with anti-FcyRIII (2.4G2) for 20 min before staining with antibodies agains specific surface markers. Stainings were performed at 4°C in HBSS supplemented with 1% FCS for 20 min. Dead cells were excluded using LIVE/DEAD® Fixable Aqua Dead Cell Stain Kit (Invitrogen). Acquisition of flow cytometry data was done using the LSR Fortessa instrument (BD Biosciences) or the LSR Symphony instrument (BD Biosciences). Data analysis was done by FlowJo 9.9.4 (Tree Star).

### NK Cell Analysis of Ca^2+^ Flux

Mouse NK cells were isolated from spleen using a negative selection kit for NK cell isolation (Miltenyi Biotec), which results in a NK cell purity of >90%. Purified NK cells were subsequently incubated with antibodies against cell surface receptors. After antibody staining, NK cells were stained for 20 min at 4°C in HBSS + 1% FCS and probenecid with calcium dyes Fluo-4 and Fura-red (Life Technologies). Cells were then kept on ice until analysis.

Primary human NK cells were purified from peripheral blood mononuclear cells (PBMC) from healthy volunteers by a similar negative isolation procedure as for mouse cells, according to the manufacturer's instructions (Miltenyi Biotec). Human NK cells were rested overnight in RPMI1640 (Gibco) supplemented with 10% fetal calf serum (Sigma-Aldrich) in a humidified incubator at 37°C and 5% CO_2_ (v/v). A viability check for 90–95% live cells was performed by a counter. Rested NK cells were washed once with assay buffer (HBSS supplemented with 1 mM probenecid (Gibco and ThermoFisher) and one million cells per condition transferred to a 96-well V-bottom plate (Sarstedt). Cells were then simultaneously labeled with fluorochrome conjugated and indicated concentrations of biotinylated monoclonal antibodies as well as loaded with 1 μM of the calcium indicator Fluo-8 a.m. (Abcam) in assay buffer for 1 h on ice. Afterwards, cells were washed, resuspended in cold assay buffer and kept on ice until acquisition on an LSR II Fortessa (BD Biosciences).

To record Ca^2+^ flux, tubes were pre-warmed at 37°C in a water bath for 10 min after which a baseline fluorescence signal was acquired for 30 s. Following this period, the tube was removed from the cytometer and a crosslinker was added. For mouse NK cells, 6 μl of polyclonal purified Goat-anti-Mouse IgG or Donkey-anti-Rat IgG (Jackson Immunoresearch), both at 1,3 mg/ml, was added to a final concentration of 20 μg/ml. For human NK cells, 1 μg of streptavidin was added, to a final concentration of 3 μg/ml to induce cross-linking of biotinylated antibodies. After the addition of crosslinker, acquisition was continued for an additional 210 s (humans) or 300 s (mouse). For human experiments and some mouse experiments, Fluo-4 (B-530-A) of Fluo-8 alone was used to record calcium flux. For some mouse experiments, both Fluo-4 (B-530-A) and Fura-red (B-710-A) were used and a ratiometric analyis (B-530-A/B-710-A) was applied during analysis.

### Analysis of Ca^2+^ Flux Data

To quantify receptor-induced iCa^2+^ mobilization, we used the kinetics analysis in the FlowJo analysis software (BD Biosciences) to determine the area under the curve (AUC) of each calcium flux plot. To allow for inter-experimental comparison, we used the kinetics algorithm in FlowJo to divide each calcium plot into 16 arbitrary regions and determined the AUC for each of those. When 16 regions were used, the first 2 always defined the time before addition of crosslinker. This allowed us to use the mean AUC value from these two regions as background flourescence, which was subsequently subtracted from the AUC value of each of the remaining regions, allowing the creation of an AUC curve reflecting the specific enhancing effect of the crosslinker relative to the background for each plot. In doing so, inter-experimental variation was reduced and a combined analysis between experiments became feasible. Region 3, encompassing the gap in the curve resulting from removal of the tube for addition of crosslinker, was excluded from the analysis. AUC values were exported from FlowJo into MS-Excel where calculations were performed.

### Inhibitory Receptor Crosslinking and Effect on Ca^2+^ Flux

To measure the influence of inhibitory crosslinking on activating receptor signaling, purified NK cells were stained with fluorescently labeled antibodies against inhibitory receptors together with a live/dead stain, a flourescently tagged detection antibody for NK cells (to which the crosslinker did not bind), Fluo-4/Fura-red and a triggering antibody for NK cells. Analysis of Ca^2+^ flux was performed as described above in the previous sections. During analysis, inhibitory receptor-positive and negative NK cells were separately gated based on their flourescence and analyzed with kinetics parameter for ratio (Fluo-4/Fura-red) and AUC (Area Under the Curves) as described in the previous sections. For some summary experments (e.g., in Figure 2) an “inhibition index” was calculated, which indicates the AUC for inhibitory receptor-positive NK cells over the AUC for inhibitory receptor-negative NK cells. The lower the figure, the larget the difference and the better the inhibition.

### Priming of NK Cells Using IL-15

NK cells were incubated with recombinant IL-15 (ImmunoTool, Germany) for 2 h at 37°C to a final concentration of 100 ng/ml. Control cells were incubated at 37°C in the absence of IL-15. Following priming, cells were washed to remove unbound IL-15 after which they were incubated with antibodies to cell surface receptors and with calcium dyes. Ca^2+^ flux was analyzed as described in the previous sections.

### Statistical Analysis

Statistical analysis was performed using GraphPad Prism 5. A paired Student's *T*-test was used for some experiments. For other experiments, we used a one-way Anova with multiple comparisons and Tukey's correction, as indicated in the figure legends. The degree of significance is indicated as follows: ^*^P < 0.05, ^**^P < 0.01, ^***^p < 0.001, ns, not significant.

## Author Contributions

SG performed mouse experiments, analyzed data, and wrote the manuscript. PH analyzed data, wrote the manuscript, and provided funding for the study.

### Conflict of Interest Statement

The authors declare that the research was conducted in the absence of any commercial or financial relationships that could be construed as a potential conflict of interest.

## References

[1] Lam VC Lanier LL. NK cells in host responses to viral infections. Curr Opin Immunol. (2017) 44:43–51. 10.1016/j.coi.2016.11.003 27984782 PMC5451301

[2] Spits H Bernink JH Lanier L. NK cells and type 1 innate lymphoid cells: partners in host defense. Nat Immunol. (2016) 17:758–64. 10.1038/ni.3482 27328005

[3] Molgora M Bonavita E Ponzetta A Riva F Barbagallo M Jaillon S. IL-1R8 is a checkpoint in NK cells regulating anti-tumour and anti-viral activity. Nature (2017) 551:110–4. 10.1038/nature24293 29072292 PMC5768243

[4] Screpanti V Wallin RP Grandien A Ljunggren HG. Impact of FASL-induced apoptosis in the elimination of tumor cells by NK cells. Mol Immunol. (2005) 42:495–9. 10.1016/j.molimm.2004.07.033 15607805

[5] Lee J Dieckmann NMG Edgar JR Griffiths GM Siegel RM. Fas Ligand localizes to intraluminal vesicles within NK cell cytolytic granules and is enriched at the immune synapse. Immun Inflamm Dis. (2018). 6:312–21. 10.1002/iid3.219 29642281 PMC5946154

[6] Feuerer M Shen Y Littman DR Benoist C Mathis D. How punctual ablation of regulatory T cells unleashes an autoimmune lesion within the pancreatic islets. Immunity (2009) 31:654–64. 10.1016/j.immuni.2009.08.023 19818653 PMC2998796

[7] Hanke T Raulet DH. Cumulative inhibition of NK cells and T cells resulting from engagement of multiple inhibitory Ly49 receptors. J Immunol (2001) 166:3002–7. 10.4049/jimmunol.166.5.3002 11207249

[8] Kaplan A Kotzer S Almeida CR Kohen R Halpert G Salmon-Divon M. Simulations of the NK cell immune synapse reveal that activation thresholds can be established by inhibitory receptors acting locally. J Immunol. (2011) 187:760–73. 10.4049/jimmunol.1002208 21690326

[9] Lanier LL. Up on the tightrope: natural killer cell activation and inhibition. Nat Immunol. (2008) 9:495–502. 10.1038/ni1581 18425106 PMC2669298

[10] Guethlein LA Norman PJ Hilton HG Parham P. Co-evolution of MHC class I and variable NK cell receptors in placental mammals. Immunol Rev. (2015) 267:259–82. 10.1111/imr.12326 26284483 PMC4587382

[11] Brooks AG Posch PE Scorzelli CJ Borrego F Coligan JE. NKG2A complexed with CD94 defines a novel inhibitory natural killer cell receptor. J Exp Med. (1997) 185:795–800. 10.1084/jem.185.4.795 9034158 PMC2196137

[12] Vance RE Kraft JR Altman JD Jensen PE Raulet DH. Mouse CD94/NKG2A is a natural killer cell receptor for the nonclassical major histocompatibility complex (MHC) class I molecule Qa-1(b). J Exp Med. (1998) 188:1841–8. 10.1084/jem.188.10.1841 9815261 PMC2212405

[13] Sternberg-Simon M Brodin P Pickman Y Onfelt B Karre K Malmberg KJ. Natural killer cell inhibitory receptor expression in humans and mice: a closer look. Front Immunol. (2013) 4:65. 10.3389/fimmu.2013.00065 23532016 PMC3607804

[14] Binstadt BA Brumbaugh KM Dick CJ Scharenberg AM Williams BL Colonna M. Sequential involvement of Lck and SHP-1 with MHC-recognizing receptors on NK cells inhibits FcR-initiated tyrosine kinase activation. Immunity (1996) 5:629–38. 10.1016/S1074-7613(00)80276-9 8986721

[15] Stebbins CC Watzl C Billadeau DD Leibson PJ Burshtyn DN Long EO. Vav1 dephosphorylation by the tyrosine phosphatase SHP-1 as a mechanism for inhibition of cellular cytotoxicity. Mol Cell Biol. (2003) 23:6291–9. 10.1128/MCB.23.17.6291-6299.2003 12917349 PMC180957

[16] Matalon O Fried S Ben-Shmuel A Pauker MH Joseph N Keizer D. Dephosphorylation of the adaptor LAT and phospholipase C-gamma by SHP-1 inhibits natural killer cell cytotoxicity. Sci Signal (2016) 9:ra54. 10.1126/scisignal.aad6182 27221712

[17] Matalon O Barda-Saad M. Cbl ubiquitin ligases mediate the inhibition of natural killer cell activity. Commun Integr Biol. (2016) 9:e1216739. 10.1080/19420889.2016.1216739 28042374 PMC5193043

[18] Long EO Kim HS Liu D Peterson ME Rajagopalan S. Controlling natural killer cell responses: integration of signals for activation and inhibition. Annu Rev Immunol. (2013) 31:227–58. 10.1146/annurev-immunol-020711-075005 23516982 PMC3868343

[19] Almeida CR Davis DM. Segregation of HLA-C from ICAM-1 at NK cell immune synapses is controlled by its cell surface density. J Immunol. (2006) 177:6904–10. 10.4049/jimmunol.177.10.6904 17082605

[20] Bryceson YT March ME Ljunggren HG Long EO. Activation, coactivation, and costimulation of resting human natural killer cells. Immunol Rev. (2006) 214:73–91. 10.1111/j.1600-065X.2006.00457.x 17100877 PMC3845883

[21] Ganesan S Luu TT Chambers BJ Meinke S Brodin P Vivier E The Abl-1 kinase is dispensable for NK cell inhibitory signalling and is not involved in murine NK cell education. Scand J Immunol. (2017) 86:135–42. 10.1111/sji.12574 28605050 PMC5568956

[22] Back J Angelov GS Mariuzza RA Held W. The interaction with H-2D(d) in cis is associated with a conformational change in the Ly49A NK cell receptor. Front Immunol. (2011) 2:55. 10.3389/fimmu.2011.00055 22566845 PMC3342051

[23] Held W Mariuzza RA. Cis-trans interactions of cell surface receptors: biological roles and structural basis. Cell Mol Life Sci. (2011) 68:3469–78. 10.1007/s00018-011-0798-z 21863376 PMC11115084

[24] Scarpellino L Oeschger F Guillaume P Coudert JD Levy F Leclercq G. Interactions of Ly49 family receptors with MHC class I ligands in trans and cis. J Immunol. (2007) 178:1277–84. 10.4049/jimmunol.178.3.1277 17237373

[25] Brodin P Lakshmikanth T Karre K Hoglund P. Skewing of the NK cell repertoire by MHC class I via quantitatively controlled enrichment and contraction of specific Ly49 saubsets. J Immunol. (2012) 188:2218–26. 10.4049/jimmunol.1102801 22287714

[26] Andersson KE Williams GS Davis DM Hoglund P. Quantifying the reduction in accessibility of the inhibitory NK cell receptor Ly49A caused by binding MHC class I proteins in cis. Eur J Immunol. (2007) 37:516–27. 10.1002/eji.200636693 17236237

[27] Lucas M Schachterle W Oberle K Aichele P Diefenbach A. Dendritic cells prime natural killer cells by trans-presenting interleukin 15. Immunity (2007) 26:503–17. 10.1016/j.immuni.2007.03.006 17398124 PMC2084390

[28] Luu TT Ganesan S Wagner AK Sarhan D Meinke S Garbi N. Independent control of natural killer cell responsiveness and homeostasis at steady-state by CD11c^+^ dendritic cells. Sci Rep. (2016) 6:37996. 10.1038/srep37996 27905484 PMC5131354

[29] Marcais A Cherfils-Vicini J Viant C Degouve S Viel S Fenis A. The metabolic checkpoint kinase mTOR is essential for IL-15 signaling during the development and activation of NK cells. Nat Immunol. (2014) 15:749–57. 10.1038/ni.2936 24973821 PMC4110708

[30] Mao Y van Hoef V Zhang X Wennerberg E Lorent J Witt K. IL-15 activates mTOR and primes stress-activated gene expression leading to prolonged antitumor capacity of NK cells. Blood (2016) 128:1475–89. 10.1182/blood-2016-02-698027 27465917 PMC5025899

[31] Wang Y Zhang Y Yi P Dong W Nalin AP Zhang J. The IL-15-AKT-XBP1s signaling pathway contributes to effector functionsand survival in human NK cells. Nat Immunol. (2019) 20:10–7. 10.1038/s41590-018-0265-1 30538328 PMC6293989

[32] Lopez-Verges S Milush JM Pandey S York VA Arakawa-Hoyt J Pircher H. CD57 defines a functionally distinct population of mature NK cells in the human CD56dimCD16^+^ NK-cell subset. Blood (2010) 116:3865–74. 10.1182/blood-2010-04-282301 20733159 PMC2981540

[33] Carr WH Pando MJ Parham P. KIR3DL1 polymorphisms that affect NK cell inhibition by HLA-Bw4 ligand. J Immunol. (2005) 175:5222–9. 10.4049/jimmunol.175.8.5222 16210627

[34] Brodin P Karre K Hoglund P. NK cell education: not an on-off switch but a tunable rheostat. Trends Immunol. (2009) 30:143–9. 10.1016/j.it.2009.01.006 19282243

[35] Joncker NT Fernandez NC Treiner E Vivier E Raulet DH. NK cell responsiveness is tuned commensurate with the number of inhibitory receptors for self-MHC class I: the rheostat model. J Immunol. (2009) 182:4572–80. 10.4049/jimmunol.0803900 19342631 PMC2938179

[36] Beziat V Liu L Malmberg JA Ivarsson MA Sohlberg E Bjorklund AT. NK cell responses to cytomegalovirus infection lead to stable imprints in the human KIR repertoire and involve activating KIRs. Blood (2013) 121:2678–88 10.1182/blood-2012-10-459545 23325834 PMC3617633

[37] Bryceson YT Ljunggren HG Long EO. Minimal requirement for induction of natural cytotoxicity and intersection of activation signals by inhibitory receptors. Blood (2009) 114:2657–66. 10.1182/blood-2009-01-201632 19628705 PMC2756125

[38] Kase A Johansson MH Olsson-Alheim MY Karre K Hoglund P. External and internal calibration of the MHC class I-specific receptor Ly49A on murine natural killer cells. J Immunol. (1998) 161:6133–8. 9834098

[39] Back J Chalifour A Scarpellino L Held W. Stable masking by H-2Dd cis ligand limits Ly49A relocalization to the site of NK cell/target cell contact. Proc Natl Acad Sci USA. (2007) 104:3978–83. 10.1073/pnas.0607418104 17360463 PMC1820694

[40] Ljunggren HG Karre K. In search of the 'missing self': MHC molecules and NK cell recognition. Immunol Today (1990) 11:237–44. 10.1016/0167-5699(90)90097-S 2201309

[41] Johansson S Johansson M Rosmaraki E Vahlne G Mehr R Salmon-Divon M Natural killer cell education in mice with single or multiple major histocompatibility complex class I molecules. J Exp Med. (2005) 201:1145–55. 10.1084/jem.20050167 15809355 PMC2213126

