# Supplementary material for: Inhibitory Receptor Crosslinking Quantitatively Dampens Calcium Flux Induced by Activating Receptor Triggering in NK Cells
Source: Front Immunol. 2019 Jan 14;9:3173. doi: 10.3389/fimmu.2018.03173 (PMC6339929; doi:10.3389/fimmu.2018.03173)
Supplement: Supplementary file 1 [file Data_Sheet_1.PDF]

Table S1. Antibodies/dyes used for mouse NK cell analysis.

Clone	Species	Specificity	Fluorochrome	Cross-reactivity	Catalogue number/company	Nature of antibody	Stock concentration
PK136	Mouse	Mouse NK1.1	Purified	Not applicable	14-5941-85 Ebioscience	IgG2a, κ	0.5 mg/ml
			Alexa 700	Not applicable	108730 Biolegend	IgG2a, κ	0.5 mg/ml
			APC	Not applicable	108710 Biolegend	IgG2a, κ	0.2mg/ml
29A1.4	Rat	Mouse NKp46	Alexa 700	Not applicable	561169 BD Biosciences	IgG_{2a}, κ	0.2 mg/ml
			APC	Not applicable	137608 Biolegend	IgG2a, κ	0.2 mg/ml
			PECy7	Not applicable	137618 Biolegend	IgG2a, κ	0.2mg/ml
YE1/48.10.6	Rat	Mouse Ly49A	Pacific Blue	Not applicable	116810 Biolegend	IgG2a, κ	0.5 mg/ml
16A11	Mouse	Mouse NKG2A	APC	Not applicable	142808 Biolegend	IgG2b, κ	0.2 mg/ml
4D11	Rat	Mouse Ly49G2	APC	Not applicable	555316 BD Biosciences	IgG2a, κ	0.2 mg/ml
34-2-12	Mouse	Mouse H2-Dd	PE	Not applicable	110607 Biolegend	IgG2a, κ	0.2 mg/ml
Not applicable	Goat	Mouse IgG (H+L)	Not applicable	Min x-react to Human, Bovine Horse	115-006-062 Jackson Immunoresearch	F(ab)2	1.2 mg/ml
Not applicable	Donkey	Rat IgG (H+L)	Not applicable	Min x-react to Bovine, Chicken, Goat, Guinea Pig, Syrian Hamster, Horse, Human, Mouse, Rabbit, Sheep	712-005-153 Jackson Immunoresearch	IgG	1.3 mg/ml
		Fluo-4			Thermo Fisher LifeTechnologies F14201		50ug/40ul. For staining 0.5ug.
		Fura-Red			Thermo Fisher LifeTechnologies F3021		50ug/35ul For staining 1.42ug.
		Aqua live/dead			Thermo Fisher LifeTechnologies L34957		1:1000
		Zombie aqua live/dead			Biolegend 423101		1:100

Table S1 cont'd. Dilutions/concentrations of mouse antibodies:

Specificity	Fluorochrome	Catalogue number/company	Stock concentration	Used dilution	Used concentration
Mouse Ly49A	Pacific Blue	116810 Biolegend	0.5 mg/ml	1:100	5 μg/ml
				1:200	2,5 μg/ml
				1:400	1,25 μg/ml
				1:800	0,625 μg/ml
				1.1600	0,312 μg/ml
				1:3200	0,156 μg/ml
				1:6400	0,078 μg/ml
				1:12800	0,039 μg/ml
Mouse NKG2A	APC	142808 Biolegend	0.2 mg/ml	1:50	4 μg/ml
Mouse Ly49G2	APC	555316 BD Biosciences	0.2 mg/ml	1:400	0,5 μg/ml
				1:800	0,25 μg/ml
				1.1600	0,125 μg/ml
				1:3200	0,0625 μg/ml
				1:6400	31,2 ng/ml
				1:12800	15,6 ng/ml
Mouse H2-Dd	PE	110607 Biolegend	0.2 mg/ml	1:50	4 μg/ml

Table S2. Antibodies/dyes used for human NK cell studies:

**Clone**	**Species**	**Specificity**	**Fluorochrome**	**Cross-reactivity**	**Catalogue number/company**	**Nature of Ab**
MOPC-21	Mouse	Not applicable	Biotin	Isotype control	Biolegend 400104	IgG1, κ
S4.1	Mouse	Human CD3	APC	Not applicable	Invitrogen MHCD0305 (discontinued)	IgG2a
NCAM 16.2	Mouse	Human CD56	BUV 395	Not applicable	BD Biosciences 563554	IgG2b, κ
HCD57	Mouse	Human CD57	Pacific blue	Not applicable	Biolegend 322316	IgM, κ
REA113	Human (rec)	Human CD94	Biotin	Not applicable	Miltenyi 130-098-966	IgG1
REA110	Human (rec)	Human CD159a (NKG2A)	PE-Vio770	Not applicable	Miltenyi 130-113-567	IgG1
REA205	Human (rec)	Human CD159a (NKG2C)	PE	Not applicable	Miltenyi 130-103-635	IgG1
		Streptavidin			Pierce 21122 Thermo Scientific	
		Fluo-8 AM			Abcam ab142773	
9E2	Mouse	Human NKp46	Biotin		Biolegend 331906	IgG1, κ

Gating strategy, mouse NK cells

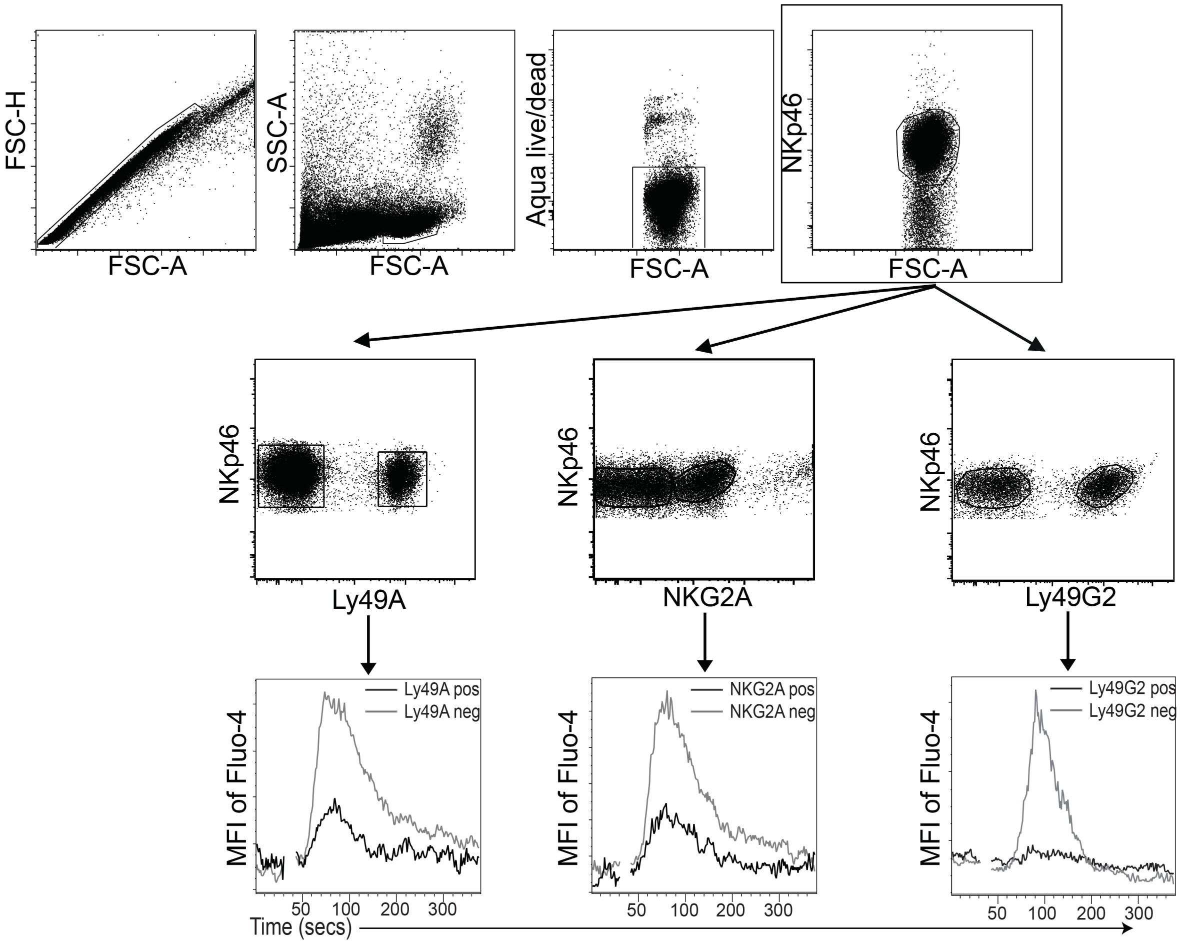

Ganesan et al., Figure S1

Gating strategy, human NK cells

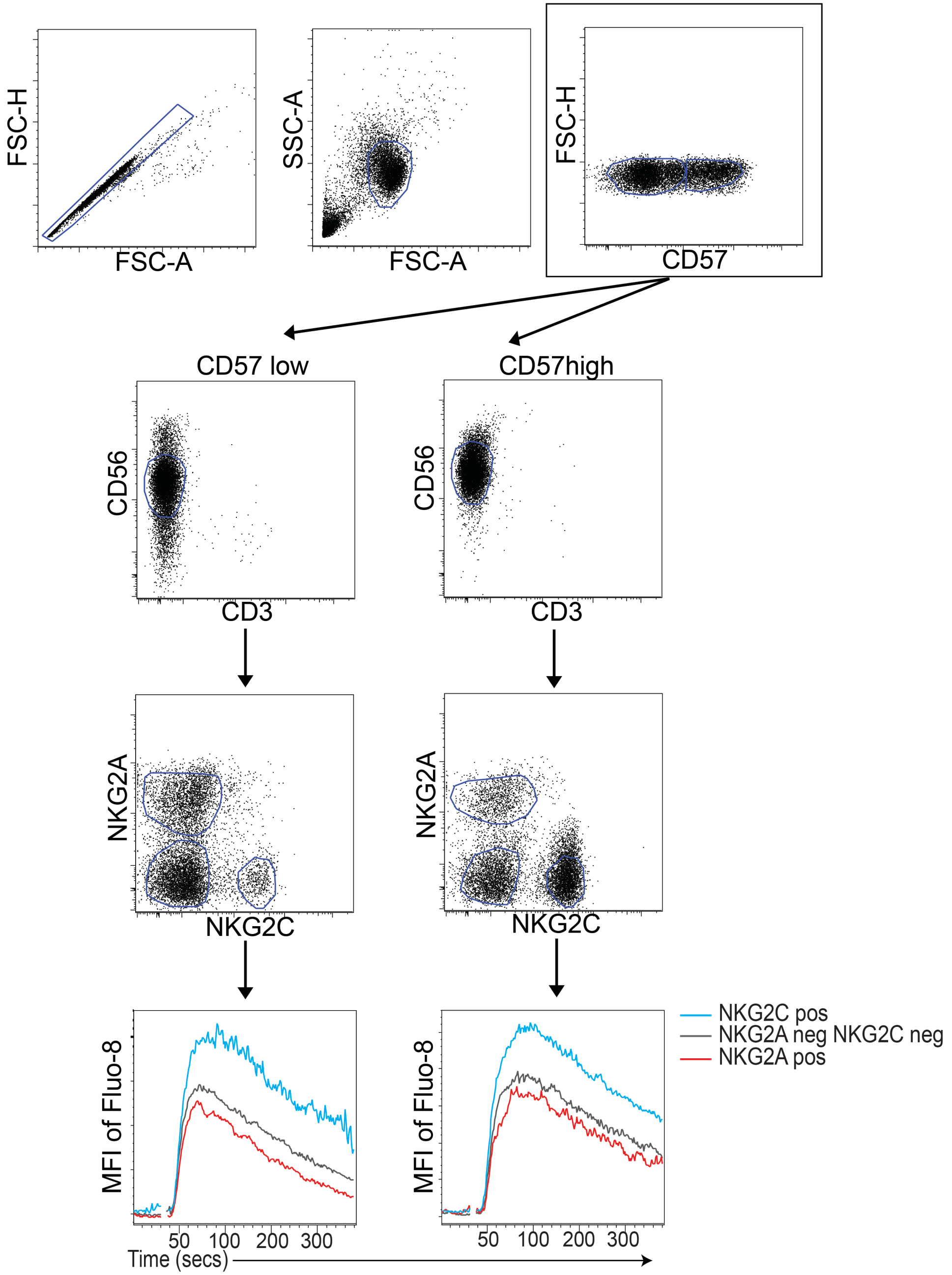

Ganesan et al., Figure S2

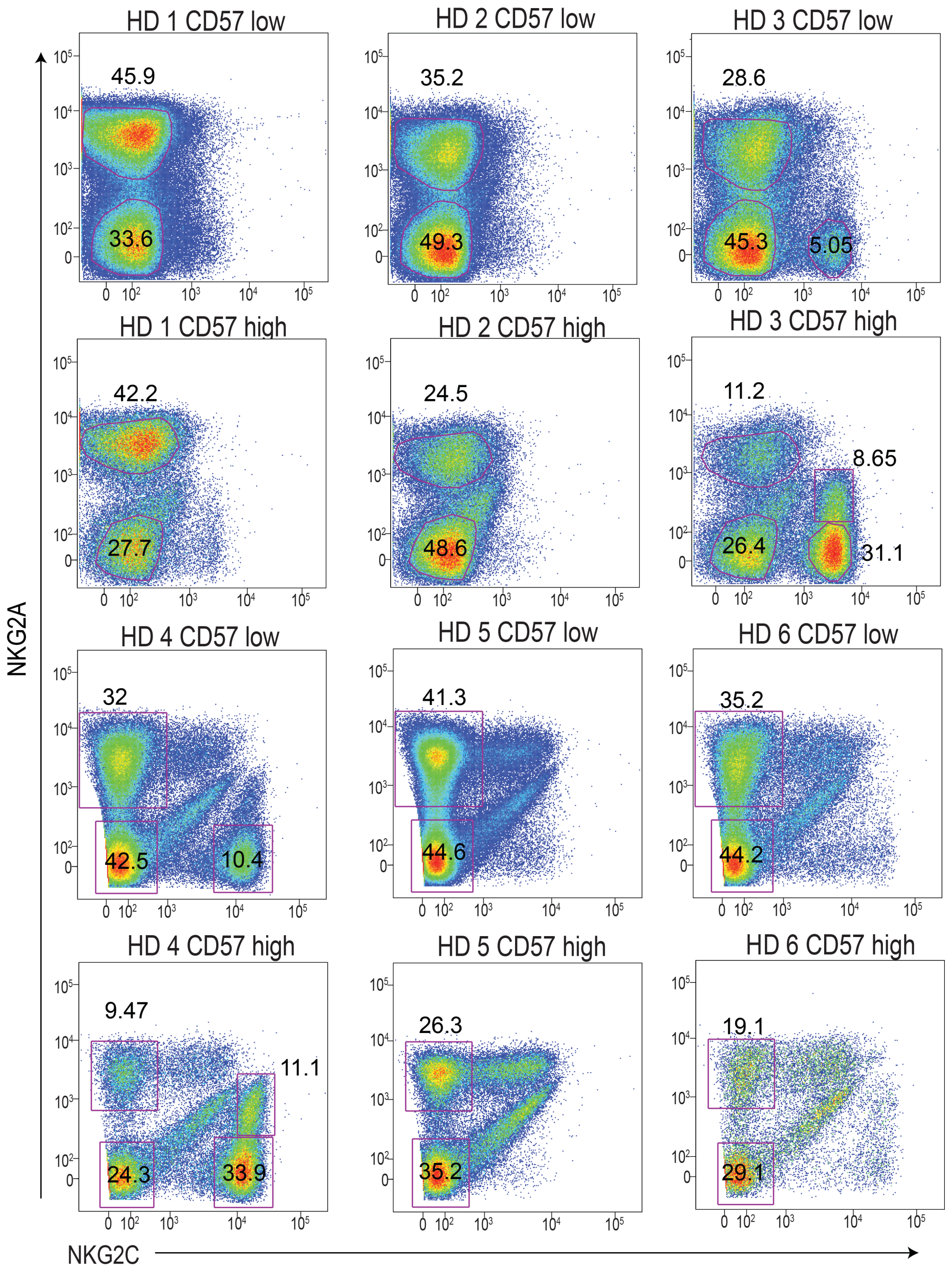

Ganesan et al., Figure S3

Figure legends Supplementary data

Figure S1. Gating strategies used to study calcium flux in mouse NK cells. Upper row: singlets were gated on lymphocytes, live cells and for NKp46 expression. Middle row: NKp46+ NK cells expressing inhibitory receptors Ly49A, NKG2A or Ly49G2 were identified in separate stainings, or together, as indicated in the main text. Lower row: Illustration of how Ca^{2+} flux can be measured in inhibitory receptor-positive and negative NK cell after co-crosslinking of the activating receptor NK1.1.

Figure S2. Gating strategy on human NK cells. Upper row: singlets were gated on lymphocytes followed by gating of the maturation marker CD57. Second row: NK cells were identified as CD56+ in CD57 low or CD57 high cells. Third row: NK cells expressing NKG2A and NKG2C were identified. Bottom row: Illustration of how Ca^{2+} flux can be measured in the subsets of NK cells in the third panel after co-crossliking of NKp46.

Figure S3. NKG2A/NKG2C stainings on purified $CD57^{high}$ and $CD57^{low}$ NK cells from 6 healthy donors (HD1-HD6).
